#  The HELENA study: Hexvix^®^-TURB vs. white-light TURB followed by intravesical adjuvant chemotherapy—a prospective randomized controlled open-label multicenter non-inferiority study

**DOI:** 10.1007/s00345-021-03719-0

**Published:** 2021-05-17

**Authors:** M. Gierth, J. Breyer, F. Zeman, H. M. Fritsche, J. Cordes, A. Karl, D. Zaak, A. Stenzl, I. Kausch von Schmeling, A. Sommerhuber, T. Zierer, M. Burger, R. Mayr

**Affiliations:** 1 Department of Urology, St. Josef Medical Center, University of Regensburg, Landshuterstrasse 65, 93053 Regensburg, Germany; 2 Center for Statistics and Clinical Studies, University of Regensburg, Regensburg, Germany; 3 Department of Urology, University Hospital of Schleswig-Holstein, Lübeck, Germany; 4 Department of Urology, Barmherzige Brüder Hospital München, Munich, Germany; 5 Department of Urology, Traunstein Medical Center, Traunstein, Germany; 6 Department of Urology, University of Tübingen, Tübingen, Germany; 7 Department of Urology, Medical Center of Westerstede, Westerstede, Germany; 8 Department of Urology, Medical Center Linz, Linz, Austria

**Keywords:** Urothelial carcinoma, Bladder cancer, Intravesical chemotherapy, Transurethral resection

## Abstract

**Purpose:**

Photodynamic diagnosis and white-light TURB with adjuvant intravesical chemotherapy (ICT) is widely used in treatment of bladder cancer. This non-inferiority trial is designed to demonstrate non-inferiority regarding recurrence-free survival (RFS) of Hexvix^®^ TURB followed by immediate instillation compared to white-light TURB with immediate instillation followed by maintenance ICT.

**Methods:**

Between 07/2010 and 12/2016, 129 patients with EORTC intermediate risk non-muscle invasive bladder cancer treated with TURB were included in this multicentre phase III study. Patients were randomized and received either white-light TURB with immediate ICT followed by maintenance ICT (*n* = 62, 20 mg Mitomycin weekly for 6 weeks as induction phase, afterwards 20 mg/month for 6 months) or Hexvix^®^ TURB with immediate ICT only (*n* = 67, 40 mg Mitomycin). Primary study endpoint was RFS after 12 months. Hexvix^®^ TURB was counted as non-inferior to white light alone if the upper limit of the one-sided 95% confidence interval of hazard ratio was lower than 1.676. Due to the non-inferiority design, the per-protocol population was used as the primary analysis population (*n* = 113)

**Results:**

Median follow-up was 1.81 years. Hexvix^®^ group showed more events (recurrence or death) than white-light group (19 vs. 10) resulting in a HR of 1.29 (upper limit of one-sided 95%-CI = 2.45; *p*_non-inferiority_ = 0.249). The ITT population yielded similar results (HR = 1.67); 3.18], *p*_non-inferiority_ = 0.493). There was no significant difference in overall survival between both groups (*p* = 0.257).

**Conclusion:**

Non-inferiority of Hexvix^®^ TURB relative to white-light TURB with maintenance Mitomycin instillation in intermediate risk urothelial carcinoma of the bladder was not proven. Hence a higher effect of maintenance ICT is to assume compared to a Hexvix^®^-improved TURB only, confirming its important role in patient treatment.

## Introduction

Bladder cancer is one of the most common urological cancers, representing the ninth and fourth most frequent tumours among women and men, respectively [1]. The current standard of care for diagnosis is white-light cystoscopy and urine cytology. Transurethral resection of bladder (TURB) is the key to establish the pathologic diagnosis and clinical stage, whereas resection of all visible tumours is recommended [2]. However, disease recurrence after TURB is remarkably common with about 30% of patients having a tumour identified at the first check cystoscopy and about 50% of patients developing a recurrence within the first year after initial TURB [3, 4]. Hence efforts to minimize this residual disease are needed.

Visualisation of bladder lesions including residual tumour tissue and carcinoma in situ during TURB can be improved using hexaminolevulinate blue light cystoscopy (HALC; Hexvix^®^, Ipsen Pharma GmbH, Ettlingen, Germany) [5–8]. Current studies showed an increasing tumour detection rate applying white-light cystoscopy plus HALC versus white-light cystoscopy alone leading HALC to be the most validated technique used today [9–11]. Nevertheless, evidence whether HALC leads to decreasing recurrence rates is still conflicting. Additionally, immediate single intravesical instillation of chemotherapy has been shown to act by destroying circulating tumour cells, and by an ablative effect on residual tumour cells at the resection site and on small overlooked tumours [12].

In a prospective trial comparing HALC plus single-shot intravesical mitomycin C and white-light assisted TURB plus single-shot mitomycin, C O`Brien and colleagues demonstrated that HALC is offering more accurate diagnostic assessment of bladder tumours, but there is no lower recurrence rate in patients treated with HALC [13]. In contrast data from a metaanalysis showed that HALC significantly improves the detection of bladder tumours leading to a reduction of cancer recurrence at 9–12 months. This is confirmed by data from Grossmann et al., finding better long-term recurrence time with a trend toward improved bladder preservation for HALC [5, 14, 15].

Treatment recommendation for low-risk bladder tumour is one immediate single instillation of intravesical chemotherapy. However, the optimal treatment strategy for intermediate risk bladder cancer patients is still debated. As a heterogeneous patient group, one immediate or maintenance intravesical instillation is recommended depending on EORTC recurrence score [12].

The intention of this prospective multicentre study is to clarify if Hexvix^®^ TURB with an immediate single intravesical mitomycin C instillation by assuming better cancer resection and lower cancer recurrence rate is not inferior to white-light TURB (WL-TURB) subsequently treated with immediate and maintenance mitomycin C intravesical chemotherapy (ICT) in patients with intermediate risk non-muscle invasive bladder cancer (NMIBC).

## Materials and methods

### Study population

The HELENA study is an institutional review board approved prospective phase III non-inferiority study with all participating sites providing the necessary data-sharing agreements. In total of 7 European University hospitals and tertiary medical centres, respectively (6 German and 1 Austrian) 247 patients were randomized from July 2010 to December 2016. Final analysis included 129 patients with intermediate risk non-muscle invasive bladder cancer. Patient drop out after randomisation and TURB was due to a post-randomisation inclusion and exclusion criteria concentrating especially on patients with intermediate risk non-muscle invasive bladder cancer. Patients received a study-specific patient information sheet and signed a consent form. All participating patients presented with clinical inconspicuous findings of the upper urinary tract documented via ultrasound and/or CT scan of the abdomen. The suspicion of bladder cancer was based on the appearance of the bladder at cystoscopy with a determined recurrence score ≥ 1 and a progression score ≤ 13, respectively, for Ta/T1 bladder cancer in accordance with EORTC risk tables [16].

### Randomisation

A randomization list stratified by centre was generated. The randomization list was included into a central online randomization tool. Investigators randomized subjects into two groups either WL-TURB with immediate ICT followed by maintenance ICT or the Hexvix^®^ TURB with immediate ICT only.

### Treatment of patients

Patients in group 1 received WL-TURB with immediate intravesical chemotherapy followed by maintenance chemotherapy (*n* = 62). Patients in group 2 received Hexvix^®^ TURB with immediate intravesical chemotherapy only (*n* = 67). Hexvix^®^ (85 mg hexylaminolevulinate in 50 mL saline) was then applicated into the bladder at least 60 min before surgery and remained 45 to 60 min.

TURB was performed by only two surgeons at each participating site. Within the immediate instillation, a 40 mg dose of mitomycin C in 40 mL saline was administered intravesically for patients in both groups. The mitomycin C retained for 45–60 min and was not administered if there was concern about bladder perforation during TURB or if there was continued bleeding. As induction chemotherapy for WL-TURB patients 3–6 weeks after TURB a weekly instillation of mitomycin C 20 mg was applicated for 6 weeks. Afterwards one monthly mitomycin C 20 mg instillation was performed for 6 months as maintenance therapy.

### Follow-up

Follow-up was identical in all patients regardless of treatment arm for 3, 6, 9 and 12 months and annual follow-up visit afterwards. It included clinical examination, ultrasound of upper urinary tract, urine cytology and white-light cystoscopy with rigid cystoscope in women and flexible cystoscope in men, respectively. In case of positive urine cytology and/or cystoscopy, Hexvix^®^ TURB was performed within 1 month. Cause of death would have been determined by the treating physician and by death certificates.

### Endpoint

Primary study endpoint was recurrence-free survival (RFS) after 12 months. Recurrence-free survival is defined as the time interval between the date of randomization and the date of recurrence or death (= target event) or the last known alive date without recurrence (i.e., patients who are alive and recurrence-free at the time of an analysis will be censored for recurrence-free survival at the time of their last contact). Recurrence was defined as urothelial cancer detected in follow-up visit and subsequently resected by TURB and verified as urothelial cancer by histopathology. In this non-inferiority trial, the hypothesis of the primary endpoint can be formulated as

$$ {\text{H}}_{0} :{\text{ HR}}_{{{\text{RFS}}\left( {{\text{Hexvix vs White}} - {\text{light}}} \right)}} {\mkern 1mu} \ge {\mkern 1mu} {1}.{\text{676 vs}}.{\text{ H}}_{{1}} :{\text{ HR}}_{{{\text{RFS}}\left( {{\text{Hexvix vs White}} - {\text{light}}} \right)}} {\mkern 1mu} < {\mkern 1mu} {1}.{676}. $$

with HR_RFS (Hexvix vs White-light)_ is defined as the hazard ratio regarding recurrence-free survival of patients treated with Hexvix^®^ TURB compared to patients treated with WL-TURB.

### Sample size considerations

Sample size was calculated using the primary endpoint, i.e. recurrence-free survival. Based on the results from Sylvester, we expected a 1-year recurrence rate of approximately 30% in intermediate risk patients treated with the standard treatment of maintenance intravesical chemotherapy in patients with risk profiles equivalent to the study inclusion criteria. Taking the results from Daniltchenko into account, a 1-year recurrence rate of approximately 60% was to be expected in untreated patients with risk profiles equivalent to the present inclusion criteria [16, 17]. The non-inferiority margin of a 15% increase in the 1-year recurrence rate was based on taking half of the observed benefit of the standard treatment over the placebo treatment. This corresponded to a hazard ratio (HR) of 1.676.

This non-inferiority margin was defined by the scientific clinical committee of this study considering that non-invasive bladder cancer is a chronic rather than a fatal disease and even expectant treatment has been advocated recently under certain circumstances.

This justification of the non-inferiority margin via clinical judgment was in accordance with ICH E9 and E10 guidelines and was in line with publications on phase III non-inferiority trials [18, 19]. Using these assumptions with an initially targeted accrual period of 12 months and a follow-up time of at least 12 months from recruiting the last patient, sample size was calculated to require a total of 93 target events and 113 patients per group to reject the null hypothesis of inferiority (HR ≥ 1.676) at a 0.05 one-sided significance level with 80% power.

### Statistical analysis

Demographical and other baseline data are summarized for all included patients using absolute and relative frequencies for categorical variables and mean (SD), median [IQR], min–max for continuous variables. The primary endpoint is presented graphically by a Kaplan–Meier (KM) plot. Median recurrence-free survival time with corresponding one-sided 95% confidence interval was estimated using the Kaplan–Meier method for each treatment group. The hazard ratio (HR) was estimated by means of the Cox proportional hazards model. Corresponding to the non-inferiority design, the hazard ratio is presented with a one-sided 95% confidence interval and the new treatment will be considered non-inferior to the standard treatment if the upper limit of the CI is below the non-inferiority margin of 1.676. The primary analysis is based on the per-protocol (PP) analysis set. A sensitivity analysis was conducted on the intention-to-treat (ITT) analysis set. The latter serves to confirm the robustness of the result. This planned sequence of PP and ITT analyses is in concordance with the ICH E9 guideline. Overall survival (OS) was analysed analogous to RFS. Further details were specified in the statistical analysis plan (SAP).

## Results

**Fig. 1:**
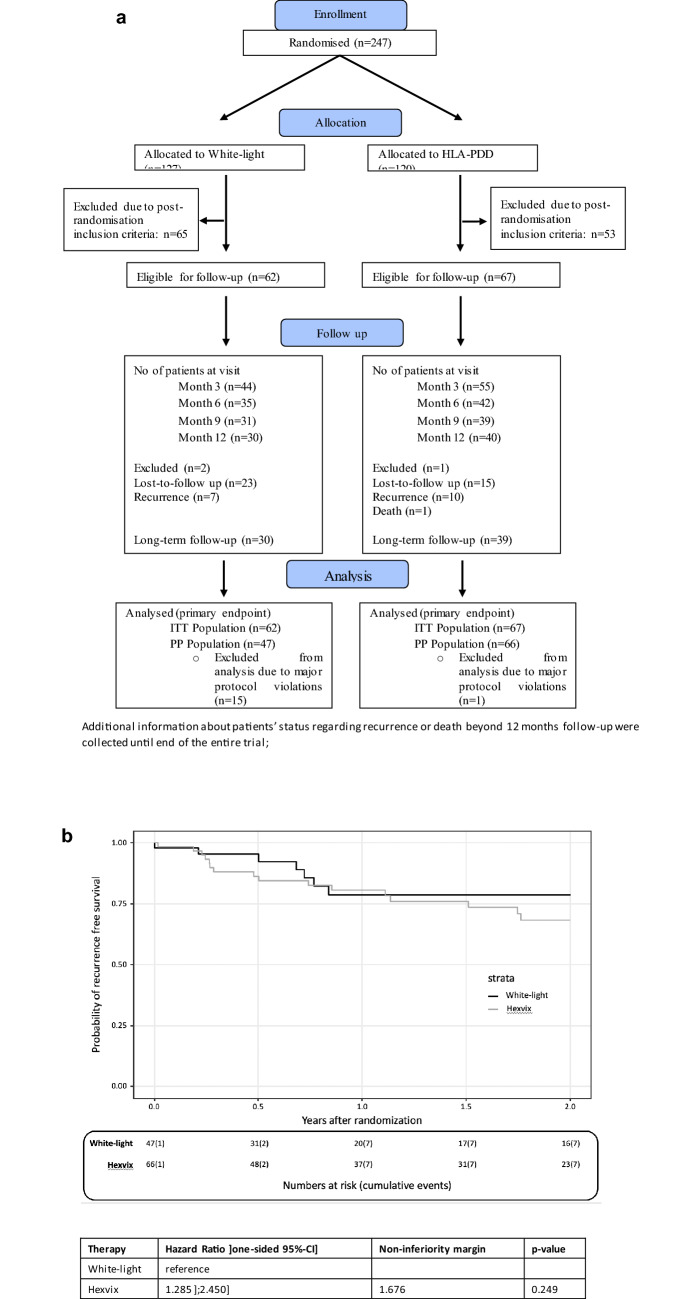
**a** Consolidated Standards of Reporting Trials (CONSORT) flow diagram demonstrating white-light and HLA-PDD study group with follow-up and analysis. Additional information about patients’ status regarding recurrence or death beyond 12 months follow-up were collected until end of the entire trial; **b** Kaplan–Meier curve and Cox proportional hazards model showing recurrence-free survival of the per-protocol population in HAL-PDD and white-light group

In total, 129 randomized patients fulfilled all inclusion and exclusion criteria and were included in the analyses. Mean age was about 69 years in both study groups, male and female patients, WHO performance status and EORTC recurrence and progression score were equally distributed between both groups. The baseline characteristics of patients and tumours are presented in Table 1. The flow of patients through the study is summarized in the Consolidated Standards of Reporting Trials (CONSORT) flow diagram (Fig. 1a).

**Table 1:** Patient and tumour characteristics of the 129 patients included and eligible for follow-up

Characteristics	Study arm	
	HAL-PDD	White light
Patients (*n*)	67	62
Age in years, mean (SD), min–max	69.7 (11.7) 42–88	69.1 (10.5) 42–83
Male/Female, *n* (%)	52/15 (77/23)	49/13 (79/21)
WHO performance status* 0/1–2, n** (%)	48/17 (71/19)	49/12 (79/21)
EORTC recurrence score, mean (SD), min–max	3.55 (1.98), Min–max 1–9	3.57 (1.72), Min–max 0–7
EORTC progression score, mean (SD), min–max	3.06 (2.19), Min–max 0–11	2.97 (1.77), Min–max 0–8
Tumour description (*n*%)		
Multifocal	10 (15)	14 (23)
Unifocal	57 (85)	48 (77)

*WHO performance status [20]

**3 patients missing

101 patients had a documentation of the TURB details, while for 28 patients no information were available. Overall 196 biopsies were documented, 105 in the Hexvix^®^ arm and 89 in the white-light arm. The histology of resected tumour is presented in Table 2.

**Table 2:** Histological analysis of resected tumour of the 129 patients included and eligible for follow-up

	Study arm	
	HAL-PDD	White light
Total NMIBC	105*	89*
Histology of resected tumour		
pTa G1 low grade	66	45
pTa G2 low grade/high grade	28	39
pTa G3 high grade	10	3
pT1 G1/G2/G3	1	2
Primary carcinoma in situ	–	–

*More than one biopsy per patient possible

A total of 37 serious adverse events (SAEs) were documented throughout the trial whereas 6 were related to study treatment. No differences regarding number of SAEs, relationship to study treatment, severity or outcome were found between the treatment arms.

The median follow-up of the per-protocol population was 1.81 (95%-CI: 1.09, 2.59) years and 1.55 (95%-CI: 1.04, 2.43) in the ITT population. Hexvix^®^ group showed more events in the sense of recurrence or death than white-light group (19 vs. 10) resulting in a HR of 1.29 (upper limit of one-sided 95% CI = 2.45; *p*_non-inferiority_ = 0.493) in the per-protocol population (Fig. 1b). The ITT population yielded similar results (HR = 1.67, 95%-CI:];3.18], *p*_non-inferiority_ = 0.249). There is no difference in the statistical significance regarding RFS between the intention-to-treat population and the per-protocol population. In both cases, non-inferiority of Hexvix^*®*^ to white light could not be proven. Median RFS time could not be calculated since more than 50% of the patients were without an event at the end of the follow-up. There was no significant difference in overall survival in the two groups (*p* = 0.257).

## Discussion

In our HELENA-study non-inferiority of Hexvix^®^ TURB with immediate ICT to white-light TURB with immediate ICT followed by maintenance ICT for intermediate risk NMIBC has not been confirmed. Even more cancer recurrence rate in the Hexvix^®^ TURB arm was higher compared to the white-light arm.

Our findings are in agreement with the results of O`Brien and colleagues. Comparing hexaminolevulinate blue-light TURB as a more accurate diagnostic assessment of a bladder tumour to WL-TURB each followed by one immediate ICT the authors found no significant difference in tumour recurrence after 3 and 12 months. As explanation for this similar result, it was stated that any small volume disease that was missed on WL cystoscopy was treated successfully by adjuvant chemotherapy hence PDD-TURB with better resection rate could be outweighed [13]. Additionally, the effectiveness of immediate ICT was confirmed in several analyses even though the basis for the reduction in recurrence has never been formally proven [17, 21, 22].

In contrast, Gallagher et al. came to conclusion that PDD is associated with significantly reduced recurrence rates at 3 years in patients receiving PDD-TURB and WL-TURB for NMIBC, respectively. The reduction of recurrence was present most likely in high-risk tumours and less in intermediate risk tumours. Even though this study was prospective, there was no patient randomisation [23].

In our study, tumour and patient classification is based on EORTC risk stratification. Optimal treatment of intermediate risk tumours to date is still a matter of debate concerning number and duration of instillation therapy [13, 16, 24]. Taking the EAU guideline as the gold standard into account, one treatment option is instillation of chemotherapy for a maximum of 1 year [25]. Results of the HELENA study, therefore, confirmed the importance of maintenance ICT and are in accordance with the gold standard therapy. However, our data do not challenge treatment of intermediate risk tumours with Hexvix^®^ TURB in general. In fact, bladder tumour often grows with a skirt of tumour around the base which often is better visible under blue light but easily to miss under white-light resection. It is, therefore, liable that the effect of maintenance ICT on recurrence rate is more intense than the optimised resection with Hexvix^®^ alone. The basis of the reduced tumour recurrence in the white-light TURB arm with maintenance ICT could be explained by chemo-destruction of small tumours and residual tumours after incomplete TURB, respectively [17].

Regarding to overall survival data, only very few events appeared in WL group and Hexvix^®^ group. The statistical informative value of these few events is very limited of course. However, the results are in accordance with the comparatively low mortality rates of NMIBC [25]. Additionally, this trial confirms that both therapy regimes Hexvix^®^ TURB and WL-TURB with maintenance ICT can be stated as safe procedures as there were only a very small numbers of serious adverse events.

As this study was prospective randomised controlled and optimally suitable proofing non-inferiority of a treatment scheme to standard therapy there still are some limitations: patient drop out after randomisation and TURB was high due to a high occurrence of low-risk tumours not suitable for final analyses. Nevertheless, to our opinion, studying a possible benefit of a technique in the population for which it was intended to be used is of utmost importance as in the HELENA study for the intermediate risk NMIBC patients. Additionally, in a multicentre study quality of Hexvix^®^ TURB and WL-TURB can differ between participating centres even experience with Hexvix^®^ was implemented in each site long before study beginning and with high surgical value.

Unfortunately, not all patients received the maintenance ICT as foreseen in study protocol with a decreasing number of patients’ visits after 3, 6, 9 and 12 months, respectively. This leads to the possibility that the present study is not sufficiently powered to detect small differences in the outcome of the two arms. However, as the trial was initiated sample size estimations have been performed and were adjusted to the study design.

## Conclusion

The HELENA data confirm the gold standard therapy for intermediate risk NMIBC by performing maintenance instillation chemotherapy at current time. Non-inferiority of Hexvix^®^ TURB relative to WL-TURB with maintenance Mitomycin was not proven. Further studies are needed to answer the question if better resection rates of Hexvix^®^ TURB may lead to an adaption in frequency and endurance of instillation therapy.
